# Risk Factors Associated with the Severity of COVID-19

**DOI:** 10.21315/mjms2023.30.3.7

**Published:** 2023-06-27

**Authors:** Martono, Fitri Fatmawati, Sri Mulyanti

**Affiliations:** Department of Nursing, Politeknik Kesehatan Kemenkes Surakarta, Surakarta, Indonesia

**Keywords:** COVID-19, risk factor, severe, mortality, comorbidity

## Abstract

The case of coronavirus disease (COVID-19) has become a global crisis. In addition, more variants of the virus have been discovered with easier transmission and more harmful effects. Thus, understanding the risk factors associated with the susceptibility and severity of COVID-19 is critical for disease control. This review article aims to describe the risk factors associated with the severity of COVID-19. This study uses the article review method from research results obtained through searching the journal sites Google Scholar, PubMed, ProQuest and ScientDirect in the 2020–2021 period. To find articles that match the inclusion criteria we used the Preferred Reporting Items for Systematic Reviews and Meta-Analyses (PRISMA) strategy. A total of nine studies met the inclusion criteria for this review. These nine studies were assessed for quality, data extraction and synthesis. Risk factors that contribute to the severity of COVID-19 are age, gender, chronic comorbidities, cardiovascular disease, diabetes, hypertension, kidney failure, cancer and a history of smoking. New findings, unvaccinated patients are at higher risk of severity. Risk factors associated with the severity of COVID-19 include a person’s individual characteristics, co-morbidities, smoking history and unvaccinated.

## Introduction

Today the world is shocked by a new type of pneumonia which the World Health Organization (WHO) named severe acute respiratory syndrome coronavirus-2 (SARS-CoV-2) with coronavirus disease 2019 (COVID-19) as the disease. Although the pandemic has been running for more than 2 years, the new cases of COVID-19 are still not over. Based on global distribution data by WHO, the update on 8 January 2022, from 204 affected countries has reached 281,638,005 confirmed positive people and 5,152,602 people died. Meanwhile, according to the Indonesian COVID-19 Handling Task Force, data reached 4,265,666 positive people, 4,115,747 people recovered and 144,127 people died. From this very large number, it indicates that today’s pandemic is still very worrying, especially with so many variants appearing.

Various variants of the COVID-19 virus that have mutated such as the Alpha, Beta, Gamma and Delta variants have made this pandemic not end soon (1). Especially now that COVID-19 has been found with a new variant Omicron from South Africa since last 24 November 2021. In Indonesia, it was detected on 16 December 2021, and until now on 7 January 2022, according to (2) the Indonesian government recorded an additional 57 cases, bringing the total confirmation of Omicron to 318 people.

Given the increasing number of COVID-19 cases and the discovery of new variants, understanding the risk factors associated with the susceptibility and severity of COVID-19 becomes very important for disease control.

## Methods

In this article review following the Preferred Reporting Items for Systematic Reviews and Meta-Analyses (PRISMA) (1).

### Search Strategy

In this article review, the search for articles uses the search engines Google Scholar, PubMed, ProQuest and ScientDirect. Articles published in March 2020 to December 2021. When conducting searches, researchers used the keywords ‘risk factor’, OR ‘mortality’, OR ‘comorbidity’, OR ‘severe’ OR ‘COVID-19’. Articles used are national and international in Indonesian or English.

### Inclusion and Exclusion Criteria

The inclusion criteria in this study are: i) articles whose research respondents are confirmed COVID-19 patients; ii) the results of the article on the analysis of risk factors associated with the severity of COVID-19; iii) using a quasi-experimental study design or a systematic review or a randomised control or trial, a qualitative study or a cross-sectional study; iv) published in 2020 or 2021 and v) English.

### Study Selection

Study selection following the PRISMA guidelines (1). An article search by keyword found 1,466 articles; 1,444 articles were found after screening based on duplication. After this, 1,428 were eliminated for being irrelevant to the title and abstract. A total of 16 full-text articles were taken and nine articles were excluded because they did not meet the inclusion criteria. As a result, nine articles were used for an article review.

### Data Extraction

**Figure 1:**
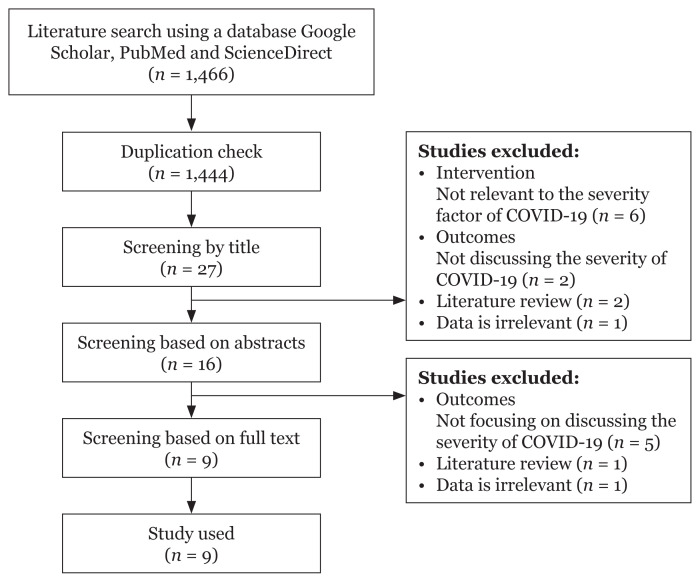
PRISMA guidelines

A total of nine studies were selected and reviewed based on inclusion criteria. The guideline used for this article review is PRISMA as shown in Figure 1. The steps taken include: i) eliminating duplication; ii) independent checking of titles, abstracts and keywords, and removing irrelevant articles according to the inclusion criteria and iii) titles and abstracts that meet the inclusion criteria and are appropriate with the aim will be selected if the text is complete. The following information is taken from the nine articles; information on demographics, study design, outcome measures, sample size, related factors, obstacles, country and year of publication.

## Results

### Study Characteristics

Of the nine studies that were used as article reviews, six of them were conducted in Asia and three was conducted in Europe, namely from London, USA, China and Malaysia. The number of samples in each article varies between 174 and 1,456,462 respondents. The studies included confirmed positive COVID-19 patients with adult age 18 years old in five studies and included all ages in four studies. The total number of respondents in the article review is 1,986,871 respondents.

### Result of Synthesis

The results of an article review show that the risk factor associated with the severity of COVID-19 patients is old age, but based on new findings, unvaccinated patients are at higher risk of severe. Male sex is also a contributing factor, as shown in Table 1. In addition, other factors are comorbidities, which consist of chronic lung disease, cardiovascular disease, diabetes mellitus, hypertension, and chronic kidney disease. The lifestyle that is included as a risk factor that plays a role is a history of smoking and unvaccinated.

## Discussion

Based on research journals (2) it shows that there are several risk factors for COVID-19 patients can experience severity when entering the hospital. These risk factors include age, gender, comorbid diseases such as hypertension, diabetes mellitus, cardiovascular disease (CVD), cancer, chronic kidney desease (CKD), chronic obstructive pulmonary disease (COPD), smoking history and unvaccinated.

### Age

Article reviews of eight research journals show seven of them are related to the severity of COVID-19 (3, 4). According to Cen et al. (5), the age factor is closely related to COVID-19 because elderly people have degenerative processes of anatomy and physiology of the body so that they are susceptible to disease and decreased immunity. Moreover, the elderly who also has comorbidities will have a weaker body so the risk to get infected with COVID-19 is higher. The function of T-cells and B-cells is attenuated with aging, and overproduction of inflammatory cytokine proteins can induce a deficiency in controlling viral replication and pro-induce a prolonged inflammatory response, thereby leading to poor outcomes. This is in line with research (6) which states that age is a risk factor for COVID-19 due to advanced age coupled with COVID-19 comorbid diseases such as hypertension.

However, based on data from COVID-19.go.id, the findings of June 2021 currently show that children are at an age that is vulnerable to get infected with COVID-19 with a percentage of 12.6%, meaning one in eight children is infected. Positive cases of COVID-19 children aged 1 year old–5 years old were 2.9%, while school-age/adolescents aged 6 years old–18 years old were 9.7%. The mortality rate for children aged 1 year old–5 years old was 0.6%, aged 6 years old – 18 years was 0.6%. According to IDAI (Indonesian Pediatric Society) in (7), one out of eight cases of COVID-19 are children, 3%–5% of them died and 50% of deaths were toddlers. This is the description of COVID-19 cases in Indonesia, especially in children, besides that, research in Rome (8) explains that children aged ≤ 18 years old have continuous symptoms (long COVID-19) if they are infected. This can occur up to more than 120 days from the initial diagnosis.

### Sex

Article reviews of eight research journals also showed that sex was associated with the severity of patients with COVID-19, especially in the group with comorbidities. According to Cen et al. (5), SARS-CoV-2 uses the angiotensin-converting enzyme 2 (ACE2) as a receptor to enter cells. The high expression of ACE2 in the testes may underlie the phenomenon that men have a higher risk of severe disease.

In addition, evidence related to male sex has a higher risk of severity than females is supported by research (9) which found that male patients (66.2%) were more prone to developing this condition critically than female patients (33.8%), possibly due to protection of the X-chromosome and sex hormones, which play important roles in innate and adaptive immunity. At the same time, men tend to be associated with poor lifestyle habits such as smoking and underlying diseases, which have been reported to be associated with an increased risk of severe COVID-19. Given that 70% of the 2,249 intensive care unit (ICU) patients in the UK are male (10) and that, of the patients who died empirically due to COVID-19 in Italy, 80% were male (11) this is consistent with the data. This suggests that hospitalised COVID-19 patients who are male and have severe disease may be at higher risk of clinical deterioration requiring ICU treatment.

### Chronic Obstructive Pulmonary Disease Comorbidity

Chronic obstructive pulmonary disease (COPD) is a comorbid disease with the greatest risk according to the odds ratio in the seventh research journal with an OR of 14.06 (12). This shows that COVID-19 patients with COPD have a risk factor of 14 times more severe than COVID-19 patients who do not have COPD. According to the pathology of the COVID-19, the coronavirus first infects the respiratory tract, hence, if there is interference in the tract, then the possibility of more severe COVID-19 symptoms is very possible (13). Patients with pneumonia and shortness of breath are also very likely to achieve poor outcomes because the chances of these patients falling into acute respiratory distress syndrome (ARDS) are increased (14).

### Cardiovascular Comorbidity

People who suffer from cardiovascular disease will be treated with drugs that contain ACE2 and angiotensin receptor blockers (ARBs) to protect the lungs. Meanwhile, SARS-CoV2 or coronavirus uses the ACE2 protein to enter cells. ACE2 is a membrane protein that has a physiological function, which is the protection of the lung, however, it becomes the entry point for viruses into the body that will cause cardiovascular toxicity. Several cardiovascular complications after being infected with COVID-19 include arrhythmia, myocarditis, acute coronary syndrome, venous thromboembolism, cardiogenic shock and heart failure (15).

In a cohort study (16) involving 191 hospitalised patients with COVID-19 in Wuhan, 48% of patients had comorbidities (67% of those who died), 30% of patients had hypertension (48% of those who died), 19% patients had diabetes (31% of those who died) and 8% of patients had coronary heart disease (24% of those who died) (SD = 17.1) with a value of 9.8 days (SD = 17.1) *P* < 0.0001.

### Diabetes Mellitus Comorbidity

In the article study, data from the first journal and the fifth journal, respectively, show an odds ratio (OR) of 3.68–1.82, meaning that patients with diabetes have 2.68–1.82 times greater risk of worsening the condition of COVID-19 if they are infected than those who do not (17). This is in line with the study of COVID-19 patients with diabetes mellitus requiring longer hospitalisation, which is 14.4 days (SD = 9.6) while without diabetes mellitus, it takes 9.8 days (SD = 17.1) with *P* < 0.0001 (18).

People with diabetes mellitus with COVID-19 will increase the secretion of hyperglycemic hormones such as catecholamines and glucocorticoids by producing glucose elevations in the blood, abnormal glucose variability and complications of diabetes (19).

Diabetes is a comorbidity for COVID-19 which is quite a lot. Diabetes mellitus is a disease of carbohydrate metabolism disorders caused by the failure of the pancreas gland to produce the hormone insulin. This condition causes high blood sugar levels and if it lasts chronically or for a long time, it can cause a decrease in the function of white blood cells or leukocytes. As a result, the immune system will decrease so that individuals will be more susceptible to infection due to the entry of microorganisms including viruses (20).

### Hypertension Comorbidity

The first and fourth journal data, respectively, show an OR of 2.72–1.44, meaning that patients with hypertension have a 2.72–1.44 times greater risk of aggravating the condition of COVID-19 patients than patients without hypertension (21). Several studies have shown that comorbid hypertension can worsen the prognosis of COVID-19 because the consumption of ACE inhibitors and ARBs as hypertension drug interventions can actually worsen COVID-19. This will worsen the condition of COVID-19 patients and increase the risk of COVID-19 morbidity and mortality. A retrospective study in China showed that COVID-19 patients with hypertension without ACE inhibitors and ARBs had reduced mortality (HR = 0.42; 95% CI: 0.19, 0.92; *P* = 0.03) (15).

### Chronic Kidney Disease Comorbidity

From a study of eight journals, there is one journal that provides data that chronic kidney failure has a risk factor for the severity of COVID-19 patients. Supported by an article (22) which stated that kidney failure patients who get infected by COVID-19, can experience worse or more severe symptoms and tend to increase the risk of mortality, especially if at the time of hospital admission the condition is moderate to severe. The author gives an opinion that patients with chronic kidney failure are related to the severity in COVID-19 patients because their immune system is not optimal, like people with COVID-19 without comorbidities, so the COVID-19 virus will be easier to develop, as a result, the other body functions decrease, this will worsen the condition of the existing patients.

### Cancer Comorbidity

In the article study of nine journals with the relation between cancer comorbidities and the severity of COVID-19 patients, the third research journal, data show that patients with cancer are more likely to have severe COVID-19 than patients without cancer. This is in line with data (23) that colorectal cancer is cancer with the third-highest incidence in the world with a total of 1.8 million cases. Colorectal cancer was also found to be the type of cancer with the highest prevalence in COVID-19 patients in other studies. There were 11 patients (29.7%) out of a total of 37 patients who had colorectal cancer. Of the 11 colorectal cancer patients with COVID-19, seven patients had severe symptoms. This means that colorectal cancer sufferers have the highest prevalence among COVID-19 patients with cancers that experience severity (24).

Based on the journals that we referenced, cancer is not an independent risk factor for the severity of patients with COVID-19 as well as if the patient underwent radiotherapy. Here’s the explanation. According to research (25) cancer patients in theseseries, cancer cannot be considered to be an independent prognostic risk factor. Recent prospective studies have shown that severe forms of COVID-19 are not directly associated with cancer and cancer treatments, but can be attributed to the many comorbidities andpoor general status of this patient population.

Supported by retrospective study (26) based on 641 patients, including 105 cancer patients, primarily designed to study the association between cancer and the risk of COVID-19-related complications, found a statistically significant association between the mortality rate of COVID-19 patients and cancer (OR = 2.34; 95% confidence interval [95% CI]: 1.15, 4.77; *P* = 0.03). This study also found a higher rate of admission in ICU (OR: 2.84; 95% CI: 1.59, 5.08; *P* < 0.01) and more severe symptoms (OR = 2.79; 95% CI: 1.74, 4.41; *P* < 0.01). In subgroup analyses, patients with lung cancer or haematological malignancies and patients receiving immunotherapy, chemotherapy or undergoingsurgery presented an excess risk of severe forms of COVID-19. However, no statistically significant relationship with severe forms of COVID-19 was observed in patients treated by radiotherapy.

Confirmed by retrospective cohort study (27) almost 25% of lung cancer patients with COVID-19 infection developed complications or died. Increasing age, stage IV disease, abnormal kidney function and low haemoglobin level were associated with a severe/fatal SARS-CoV-2 infection, while checkpoint inhibitor therapy, diabetes and abnormal creatinine levels were associated with increased mortality from COVID-19 disease.

### Smoking History

The smoking history factor is found in the first, fourth and eighth research journals. This is in line with the study (28) which stated that smoking status was related to the degree of disease severity in patients who were hospitalised in the ICU. Another study from (29) stated that smoking status was related to the severity and mortality of COVID-19 in analysis of the clinical outcame of ‘unfavourable’ the patient died or the condition became more severe. It is supported by research (30) stated that exposure to cigarette smoke increases the expression of the COVID-19 ACE2 receptor in the human respiratory tissue, which is potentially reversible. This upregulation is likely mediated by the expansion of ACE2+ secretory cells resulting from chronic smoke exposure. Several inflammatory cytokines also trigger ACE2 up-regulation, which in turn may affect ACE2 expression due to smoking-related lung inflammation. The excessive ACE2 load in the lungs of smokers may partly explain why smokers are more likely to develop severe SARSCoV-2 infection, which requires more aggressive medical intervention.

According to research journals (30) the pooled ORs of the association between smoking and severity of patients with COVID-19, on the basis of different geographical locations of the research, indicated that smoking was statistically significantly associated with COVID-19 severity in China (OR = 1.81; 95% CI: 1.52, 2.15; *I* 2 = 30.1%), USA (OR = 1.43; 95% CI: 1.21, 1.70; *I* 2 = 82.8%) and other areas (OR = 1.47; 95% CI: 1.24, 1.74; *I* 2 = 88.9%). Subgroup analysis based on median age groups showed a statistically significant association among those whose age ≥ 40 years old ( *P <* 0.001) but not in the age group of *<* 40 years old.

Based on research (31) smoking history was shown to be associated with increased severity of disease in COVID-19 patients. In addition, this association also existed for critical illness of patients with COVID-19 in ICU care, mortality and composite endpoints subgroup but was not significant for the mechanical ventilation sub-group. It is still unclear whether people with smoking history are more likely to be infected with COVID-19 disease. However, individuals with smoking history tend to be more severely influenced by COVID-19, with ACE2 being a potential explanation. This enzyme has been identified as a cell entry receptor for SARS-CoV-2. Exposure to cigarette smoke increased the expression of coronavirus receptor ACE2 in respiratory tissues of rodents and humans, which was potentially reversible, according to a study (32). This up-regulation is possibly mediated by the expansion of ACE2 and secretory cells resulted from chronic smoke exposure. Some inflammatory cytokines also trigger the up-regulation of ACE2, which may further affect the expression of ACE2 due to smoking-related pulmonary inflammation. The excessive burden of ACE2 in the lungs of smokers may partly explain why smokers are more likely to develop severe SARS-CoV-2 infection, which requires more aggressive medical intervention (32).

### Unvaccinated

According to Dr. James McCarthy (33), the hospital’s chief executive physician, around 85% of the patients at Memorial Hermann Health System’s ICUs were not vaccinated against COVID-19. In addition to the threatening severity in COVID-19 patients who have not been vaccinated, there are studies explaining the effectiveness of the third dose of BNT162b2 mRNA vaccine to prevent the severity of COVID-19 patients. Vaccine effectiveness evaluated at least 7 days after receipt of the third dose, compared with receiving only two doses at least 5 months ago, was estimated to be 93% (231 events for two doses versus 29 events for three doses; 95% CI: 88, 97) for admission to hospital, 92% (157 versus 17 events; 95% CI: 82, 97) for severe disease and 81% (44 versus seven events; 95% CI: 59, 97) for COVID-19-related death (34). From this, it is clear that vaccination has an important role to play in preventing the severity of COVID-19 patients and a solution that can be attempted in the near future as a bull to face the new type of COVID-19 variant virus is booster vaccination in patients who meet the criteria.

## Conclusion

Individual characteristic factors, among others, older age > 60 years old and male sex have a greater risk of severity compared to productive age and female sex. Meanwhile, the new finding is that children aged 18 years old have continuous symptoms (old COVID-19) if the child is confirmed positive. In addition, several comorbidities that can increase the severity of COVID-19 patients are COPD, cardiovascular disease, diabetes mellitus, hypertension, chronic kidney failure, cancer and a history of smoking. Recent findings that play a role are patients not vaccinated.

## Figures and Tables

**Table 1:** Summary of study selection COVID-19 patient severity risk factors

Author	Country	Design	Sample	Variables	Result
Zheng et al.	China	Systematic review	3,027 respondents	Risk factors for critical COVID-19 cases and deaths	Age over 65 years old (OR = 6.06; 95% CI: 3.98, 9.22); gender (OR = 1.76; 95% CI: 1.41, 2.18); history of smoking (OR = 2.51; 95% CI: 1.39, 3.32); diabetes (OR = 3.68; 95% CI: 3.68, 5.03); hypertension (OR=2.72, 95% CI: 1.60, 4.64); cardiovascular disease (OR = 5.19; 95% CI: 3.25, 8.29) and respiratory disease (OR = 5.15; 95% CI: 2.51, 10.57)
Jain et al.	London	Cohort study	1,813 respondents	Prediction of symptoms and comorbidities for severe COVID-19 patients admitted to the ICU	Age, gender, chronic obstructive pulmonary disease, CVD, hypertension
Tian et al.	China	Cohort study	751 respondents	Clinical characteristics and risk factors COVID-19 disease severity	Age, gender, cancer (OR = 3.61; 95% CI: 2.59, 5.04; *P* < 0.0001)
Li et al.	China	Cohort study	548 respondents	Risk factors for severity and death	Age > 65 years old, history of smoking, coronary heart disease, hypertension, diabetes
Cen et al.	China	Cohort study	1,007 respondents	Risk factors for the development of COVID-19 disease	Age (2.56; 95% CI: 1.97, 3.33); male gender (HR = 1.79; 95% CI: 1.41, 2.28); hypertension (HR = 1.44; 95% CI: 1.11, 1.88), diabetes (HR = 1.82; 95% CI: 1.35, 2.44); chronic obstructive pulmonary disease (HR = 2.01; 95% CI: 1.38, 2.93) and coronary artery disease (HR = 1.83; 95% CI: 1.26, 2.66)
Zhang et al.	China	Cohort study	174 respondents	Risk factors for the severity of COVID-19 patients with type 2 DM	Age, gender, diabetes with coronary heart disease, diabetes with stroke, diabetes with hypertension
Sim et al.	Malaysia	Cohort study	5,889 respondents	Clinical characteristics and risk factors for severe COVID-19 infection	Age, chronic kidney disease (11.3% versus 0.7%, *P* = 0.001); lung (3.6% versus 0.3%, *P* = 0.001); heart (14.0% versus 2.3%, *P* = 0.001); diabetes 48.6% versus 13%, *P* = 0.001)
Zhang et al.	Europe	Sistematic literature	517,020 respondents	Smoke history, severe, critical	Associating smoking history and COVID-19 severity, the pooled OR was 1.55 (95% CI: 1.41, 1.71). Smoking was significantly associated with risk of admission to the ICU (OR = 1.73; 95% CI: 1.36, 2.19), increased mortality (OR = 1.58; 95% CI: 1.38, 1.81) and critical illness composite endpoints (OR = 1.61; 95% CI: 1.35, 1.93)
Barda et al.	Israel	Cohort study	1,456,642 respondents	Third dose BNT162b2 mRNA COVID-19 vaccine, severe	Vaccine effectiveness evaluated at least 7 days after receipt of the third dose, compared with receiving only two doses at least 5 months ago, was estimated to be 93% (231 events for two doses versus 29 events for three doses; 95% CI: 88, 97) for admission to hospital, 92% (157 versus 17 events; 95% CI: 82, 97) for severe disease and 81% (44 versus seven events; 95% CI: 59, 97) for COVID-19-related death
